# Validated Stability-Indicating RP-HPLC Method for the Simultaneous Determination of Azelnidipine and Olmesartan in Their Combined Dosage Form

**DOI:** 10.3797/scipharm.1312-14

**Published:** 2014-02-27

**Authors:** Jayvadan K Patel, Nilam K Patel

**Affiliations:** ^1^ Nootan Pharmacy College, S.P. Sahkar Vidhyadham, Kamana Crossing, Visnagar 384315, Mehsana, Gujarat, India.; ^2^ Department of Pharmaceutical Sciences, Hemchandracharya North Gujarat University, Patan 384265, Gujarat, India.

**Keywords:** Azelnidipine, Olmesartan, RP-HPLC, Stability-indicating determination, Forced degradation

## Abstract

A simple, rapid, and highly selective RP-HPLC method was developed for the simultaneous determination of Azelnidipine (AZL) and Olmesartan (OLM) drug substances in the fixed dosage strength of 16 mg and 20 mg, respectively. Effective chromatographic separation was achieved using a Hypersil GOLD C18 column (150 mm × 4.6 mm internal diameter, 5 µm particle size) with a mobile phase composed of methanol, acetonitrile, and water in the ratio of 40:40:20 (by volume). The mobile phase was pumped using a gradient HPLC system at a flow rate of 0.5 mL/min, and quantification of the analytes was based on measuring their peak areas at 260 nm. The retention times for Azelnidipine and Olmesartan were about 8.56 and 3.04 min, respectively. The reliability and analytical performance of the proposed HPLC procedure were statistically validated with respect to system suitability, linearity, ranges, precision, accuracy, specificity, robustness, detection, and quantification limits. Calibration curves were linear in the ranges of 2–48 μg/mL for Azelnidipine and 2.5–60 μg/mL for Olmesartan with correlation coefficients >0.990. The proposed method proved to be selective and stability-indicating by the resolution of the two analytes from the forced degradation (hydrolysis, oxidation, and photolysis) products. The validated HPLC method was successfully applied to the analysis of AZL and OLM in their combined dosage form.

## Introduction

Azelnidipine (AZL), (±)-3-[1-(diphenylmethyl)azetidin-3-yl] 5-propan-2-yl 2-amino-6-methyl-4-(3-nitrophenyl)-1,4-dihydropyridine-3,5-dicarboxylate, is a new dihydropyridine derivative with calcium antagonistic activity [1]. The recommended dosing of AZL is 16 mg per day. A literature survey revealed that AZL is not yet official in any pharmacopoeia. Very few analytical methods have been reported for the determination of AZL, which include HPLC [2, 3], an LC-MS method [4, 5], LC-ESI-MS [6, 7], and HPLC-MS-MS [8], in which two methods were for the formulation and the remaining for human plasma. No single method discussed the stability study profile. In this study, we focus on the degradation study of Azelnidipine with Olmesartan.

Olmesartan medoxomil (OLM) is (5-methyl-2-oxo-1,3-dioxol-4-yl)methyl 4-(2-hydroxy-propan-2-yl)-2-propyl-1-{[2'-(2/-/-tetrazol-5-yl)biphenyl-4-yl]methyl}-1*H*-imidazole-5-carboxylate. Hydrochlorothiazide (HCT) is one of the oldest and most widely used thiazide diuretics with anti-hypertensive activity [9]. A literature survey revealed that OLM is not yet official in any pharmacopoeia. Several analytical methods have been reported for the determination of olmesartan medoxomil in biological fluids, which include LC-MS-MS [10], degradation product HPLC [11], HPTLC [12], and HPLC with a dissolution study [13].

Several clinical trials have proven that OLM and AZL have better therapeutic effects in essential hypertension rather than in a single dosage form [14]. There was only one first derivative spectrophotometric method reported for the simultaneous analysis [15]. No chromatographic method with a stability study was yet published for the combination dosage form. So, the aim of the present work was to develop a simple, sensitive, accurate, and precise HPLC SIAM for routine analysis. The proposed method was validated according to ICH guidelines [16].

## Results and Discussion

### Method Development and Optimization

The main objective prior to the development of a proper RP-HPLC method was to separate AZL and OLM from the formulation excipients and all degradation products. Moreover, the method should be simple enough for use in a routine quality control laboratory. Various mobile phases have been examined to achieve these specific goals. Both of the AZL and OLM (10 μg/ml each) spectra have sufficient absorption at 260 nm, which was therefore chosen for the entire study.

**Fig. 1.**
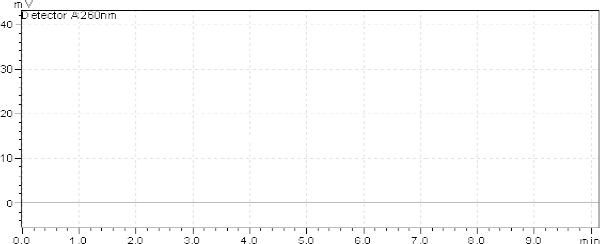
Placebo chromatogram

**Fig. 2.**
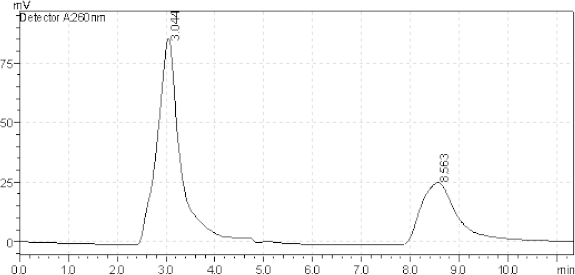
Typical chromatogram of a standard mixture of 16 μg/ml AZL (8.563 minutes), 20 μg/ml OLM (3.044 minutes).

For the study, 16 µg/ml of Azelnidipine and 20 µg/ml of Olmesartan solution were prepared for the entire standard. Different columns like the Hypersil GOLD C18 column (150 mm × 4.6 mm internal diameter, 5 µm particle size), Kromasil 100 C8 column (50 mm × 4.6 mm internal diameter, 5 µm particle size), and ACE 5 C18 column (150 mm × 4.6 mm internal diameter, 5 µm particle size) were tried for method development. The Hypersil gold column gave better results compared to the other column. In almost every system (with changes in the mobile phase) studied, Azelnidipine and Olmesartan showed retention times greater than 8.5 min and 3.0 min, respectively. Other mobile phase combinations resulted in > 8.5 min and 3.0 min, where the separation of AZL and OLM from each other and from excipients/degradation products was worse than the optimal conditions. The optimized mobile phase was composed of methanol, acetonitrile, and water in the ratio of 40:40:20 v/v/v. It was not required to change the pH of the mobile phase, as both drugs had well-resolved peaks in the optimized mobile phase. Figure 1 shows a typical chromatogram of the placebo at the optimized conditions. Figure 2 also shows a typical HPLC chromatogram of the freshly prepared mixture of AZL and OLM using the optimized conditions.

### Method Validation

After the successful optimization of the RP-HPLC method, it was validated in accordance to the ICH guidelines. Parameters such as system suitability, specificity (placebo and forced degradation interferences), sensitivity (LOD and LOQ), linearity, range, accuracy (recovery), precision (repeatability and intermediate precision), robustness, and stability-indicating capability were all validated.

### System Suitability

The system suitability was determined by injecting six replicates of the standard solutions and analyzing each active ingredient for its peak area, peak tailing factor, resolution, number of theoretical plates, and capacity factor. The system suitability results for a combined solution of 16 μg/ml AZL and 20 μg/ml OLM revealed a %RSD of less than 1.0 % for both peak areas. This method meets the accepted requirements as shown in Table 1.

**Tab. 1.** Summary of the accepted system suitability requirements

Parameter	AZL	OLM	Accepted limit
%RSD	0.30	0.23	≤ 2.0%
Tailing factor (Tf)	0.91	1.02	≤ 2.0
Resolution (Rs)	3.292	–	≥2.0
Number of theoretical plates (N)	3059	5216	≥ 3000
Capacity factor (k')	5.23	1.06	≥ 1.0

### Specificity (Placebo and Forced Degradation Interference)

**Fig. 3.**
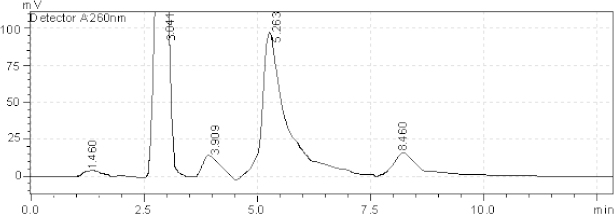
HPLC chromatogram of acidic degradation (1 N HCl) of the tablet solution after reflux 4 hr, AZL (8.460 minutes), OLM (3.041 minutes). The unknown degraded impurities appeared at 1.460, 3.909, and 5.263 minutes.

Generally, the specificity of a method is its suitability for the analysis of a compound in the presence of potential impurities. Placebo, standards, and sample test solutions were all injected at the same wavelength of 260 nm to demonstrate the specificity of the optimized method. A comparison of the retention times of AZL and OLM in sample solutions and in the standard solutions were exactly the same. Figures 2 and 3 showed that there were no interferences at the retention times for AZL and OLM due to the placebo. Therefore, the proposed method is suitable for the quantification of the active ingredients in tablet formulation.

The specificity of the method for AZL and OLM has been assessed by performing forced degradation studies on the active ingredients separately to indicate the initial results, and also on samples of the tablet formulation. The stress conditions studied were sunlight, heat (reflux), acid hydrolysis (1.0 N HCl), base hydrolysis (1.0 N NaOH), and oxidation (3% H_2_O_2_). The sample stress solutions were analyzed against freshly prepared standards and samples. The assays for the stressed standards and sample solutions were calculated as summarized in Table 2. The degradation product is listed in Table 3.

**Fig. 4.**
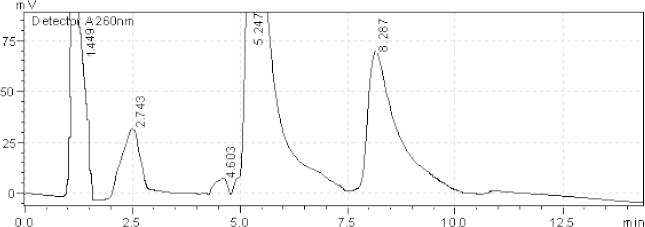
HPLC chromatogram of basic degradation (1 N NaOH) of the tablet solution after RT 6 hr, AZL (8.287 minutes), OLM (3.0 minutes). The unknown degraded impurity appeared at 1.449, 2.743, 4.603, and 5.247 minutes.

**Fig. 5.**
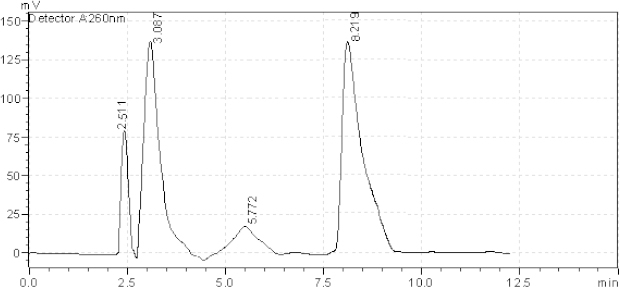
HPLC chromatogram of the tablet solution upon exposure to reflux for 12 hours, AZL (8.219 minutes), OLM (3.087 minutes). The unknown degraded impurity appeared at 2.511 and 5.772 min.

**Fig. 6.**
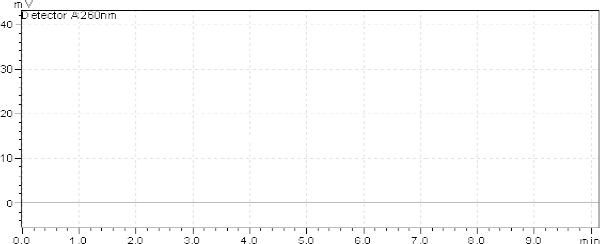
HPLC chromatogram of oxidative degradation of the tablet solution after 3 days, AZL (8.539 minutes), OLM (3.066 minutes). The unknown degraded impurity appeared at 1.593, 2.754, 4.620, and 6.270 minutes.

**Fig. 7.**
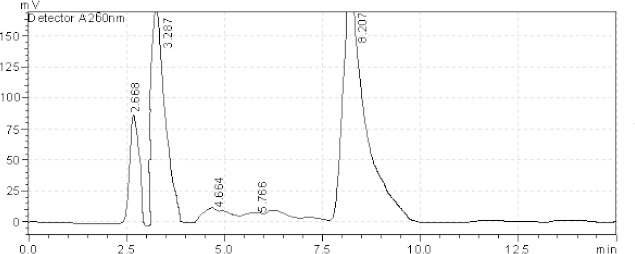
HPLC chromatogram of the tablet solution upon exposure to sunlight for 6 hours, AZL (8.207 minutes), OLM (3.287 minutes). The unknown degraded impurity appeared at 2.668, 4.664, and 5.766 minutes.

Table 2 reveals that Azelnidipine showed extensive degradation under all conditions except aqueous degradation. Olmesartan showed degradation under all conditions except sunlight and aqueous degradation. The resolution for both active ingredients was found to be greater than 2.0. Therefore, there was no interference between the main active ingredients and any other stress impurity peaks in the chromatogram. Almost the same pattern of degradation was obtained for both AZL and OLM in their samples. Figures (3–7) show the chromatographic profiles of the active ingredients and the degradation products after exposing the sample solution to different stress conditions as in Table 2.

**Tab. 2.** Summary of the forced degradation of AZL and OLM standards and tablet solution

Condition of forced degradation	% Degradation of API		% Degradation of formulation	
	AZL	OLM	AZL	OLM
1 N HCl, reflux, 4 hr	90.30	92.30	91.21	91.10
1 N NaOH, RT, 6 hr	62.74	100	60.21	100
Water, reflux, 12 hr	11.82	0	10.85	0
3 % w/v H_2_O_2_, RT, 3 days	97.73	96.80	95.65	93.87
Sunlight, summer, 6 hr	21.63	1.09	20.13	0.58

**Tab. 3.** Summary of the forced degradation product of AZL and OLM standards and solution

Condition of forced degradation	Azelnidipine		Olmesartan	
	RT of Drug	RT of Degradation Products	RT of Drug	RT of Degradation Products
1 N HCl, reflux, 4 hr	8.460	3.90, 5.26	3.04	1.46
1 N NaOH, RT, 6 hr	8.29	2.74, 4.60, 5.24	–	1.45
Water, reflux, 12 hr	8.21	2.51, 5.77	3.09	–
3% w/v H_2_O_2_, 3 days	8.53	2.75, 4.62, 6.27	3.06	1.59
Sunlight, summer, 6 hr	8.20	2.67, 4.67, 5.77	3.29	–

### Sensitivity

The sensitivity of the method was explored via measurement of the limit of detection (LOD) and limit of quantitation (LOQ) for AZL and OLM at a signal-to-noise ratio of 3 and 10, respectively. The LOD was found to be 0.41 and 0.29 μg/ml for AZL and OLM, respectively. The LOQ was found to be 1.24 and 0.89 μg/ml for AZL and OLM, respectively, with the RSD less than 2% (accepted value is less than 10 %).

### Linearity and Range

**Fig. 8.**
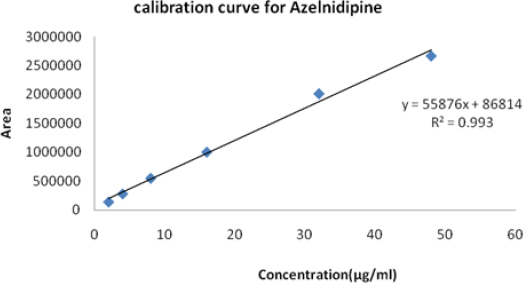
Linearity and range for AZL

**Fig. 9.**
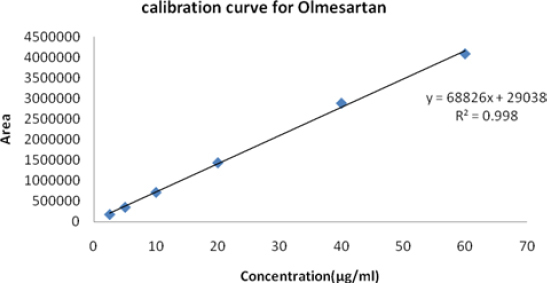
Linearity and range for OLM

The calibration curves were plotted over the concentration range of 2–48 µg/ml for Azelnidipine and 2.5–60 µg/ml for Olmesartan. Accurately measured working standard solutions of Azelnidipine (0.2, 0.4, 0.8, 1.6, 3.2, and 4.8 ml) and Olmesartan (0.25, 0.5, 1.0, 2.0, 4.0, and 6.0 ml) were transferred to a series of 10-ml volumetric flasks and the volume in each flask was adjusted to 10 ml with the mobile phase. Resulting solutions were injected into the column and the peak areas obtained at the retention times 3.04 and 8.56 minutes at a flow rate of 0.5 ml/min were measured at 260 nm for Olmesartan and Azelnidipine, respectively. Calibration curves were constructed by plotting peak area versus concentration. Each reading was the average of three determinations (figures 8 and 9). Regression analysis data is shown in Table 4.

**Tab. 4.** Regression statistics

Active ingredient	Linearity range (μg/ml)	(R2)	Linearity equation^*^
AZL	2–48	0.993	Y = 55876X + 86814
OLM	2.5–60	0.998	Y = 68826X + 29038

^*^ Y is the peak area and X is the concentration.

### Accuracy (Recovery)

Accuracy was determined by the recovery study of known amounts of AZL and OLM standards added to a sample solution. Different concentrations of the two active ingredients were added to the sample and the recovery was measured. The data obtained for the evaluation of linearity were used. The accuracy as reflected from recovery data and statistical evaluation of the assay for the two active ingredients is listed in Table 5. The average recovery data of AZL and OLM showed results between 99.36% and 101.07% with the %RSD of less than 1.75%, which are within acceptable limits (98.0 to 102.0% recovery and %RSD of no more than 2.0%).

**Tab. 5.** Average recoveries, %RSD values at five concentration levels of spiking with AZL and OLM

Drug	Amount taken (μg/ml)	Amount added (μg/ml)	Amount found (μg/ml) ± SD (n=3)	% Recovery ± SD (n=3)
Azelnidipine	16	50%	7.95 ± 0.08	99.36 ± 1.05
		100%	16.02 ± 0.06	100.16 ± 0.40
		150%	24.26 ± 0.08	101.07 ± 0.35
Olmesartan	20	50%	10.00 ± 0.17	100.04 ± 1.75
		100%	20.11 ± 0.21	100.57 ± 1.03
		150%	30.18 ± 0.29	100.61 ± 0.98

### Precision

#### Repeatability

The method precision of the instrument was checked by repeatedly injecting (n=6) the standard solution. The assay results and statistical evaluation for the assay of the two active ingredients showed %RSD values of 0.23 % and 0.30 % for AZL and OLM, respectively, which are within the acceptable limit of 2.0 %.

#### Intermediate Precision (Ruggedness)

Intermediate precision was evaluated in terms of intraday and interday precision by analyzing three different concentrated solutions three times on the same day and on different days over the entire concentration range for both drugs. The assay results and statistical evaluation for the assay of the two active ingredients revealed %RSD values of intraday 0.33–0.52 and 0.32–0.51%, interday 0.76–1.54% and 0.56–1.06%, for AZL and OLM, respectively, which are within the acceptable limit of 2.0%.

#### Robustness

Predetermined variations were performed under the experimental conditions of the RP-HPLC method to assess its robustness. The variations imposed on the chromatographic method are summarized in Table 6. The modifications include different mobile phase flow rates of (± 0.1 ml/min) and different column temperatures in the range (± 2°C). Different mobile phase compositions (in the range of ± 1 of the nominal value) and wavelength variations (± 1 nm) were also investigated. The %RSD values showed no significant changes in the final assay results of each of the above two ingredients using variations (Table 6).

**Tab. 6.** Robustness testing of the two active ingredients of AZL and OLM

Parameter	Modification	%Recovery ± SD (n=6)	
		Azelnidipine	Olmesartan
Flow rate (0.5 ml/min)	±0.1	101.32±1.21	100.42±1.11
Mobile phase composition			
Methanol:acetonitrile:water (40:40:20 v/v)	± 1	101.04±1.19	99.47±1.27
Wavelength (260 nm)	± 1	100.34±1.58	99.27±1.50
Injection volume (20 µl)	± 1	100.03±1.82	99.66±1.49
Column temperature (40°C)	± 2	100.40±1.37	100.75±1.56

#### Applicability of the Method to a Marketed Product

It is evident from the results obtained that the validated method gave satisfactory results with respect to the analysis of both drugs. The validated method was applied to a laboratory prepared tablet as shown in Table 7.

This acceptable value indicated the applicability of the proposed method for the routine quality control of the tablet formulation without interference from the excipients. This was evidenced by the good labeled claim percentages as well as the absence of any peaks in the chromatogram of the placebo.

**Tab. 7.** Results of the market product

Azelnidipine			Olmesartan		
Label claim (mg)	Amount found (mg) ± SD (n=3)	% Assay ± SD (n=3)	Label claim (mg)	Amount found (mg) ± SD (n=3)	% Assay ± SD (n=3)
16	16.11 ± 0.15	100.69 ± 0.93	20	20.22 ± 0.11	101.11 ± 0.60

### Applicability of the Method to a Marketed Product

It is evident from the results obtained that the validated method gave satisfactory results with respect to the analysis of both drugs. The validated method was applied to a laboratory prepared tablet as shown in Table 7.

This acceptable value indicated the applicability of the proposed method for the routine quality control of the tablet formulation without interference from the excipients. This was evidenced by the good labeled claim percentages as well as the absence of any peaks in the chromatogram of the placebo.

## Experimental

### Materials

The reference standards of Azelnidipine and Olmesartan were purchased from Chitichem, Rajkot. HPLC grade methanol, acetonitrile solvents, water, hydrochloric acid fuming (37%), sodium hydroxide pellets, and hydrogen peroxide (30%), were purchased from Merck (Germany).

### HPLC System

The gradient high-pressure liquid chromatography system (Shimadzu LC-2010C HT) with variable wavelength programmable UV/Vis detector, Shimadzu (Kyoto, Japan) was used for HPLC analysis. A UV-1800 double beam UV-Vis spectrophotometer, attached with computer operated software, a UV probe 2.0 with a spectral width of 2 nm, wavelength accuracy of 0.5 nm, and a pair of 1-cm matched quartz cells, was used to measure the absorbance of the resulting solutions. The Sartorius CP224S analytical balance (Göttingen, Germany) and ultrasonic cleaner (Frontline FS 4, Mumbai, India) were used during the study.

### Chromatographic Conditions

The HPLC experimental conditions were optimized on the Hypersil GOLD C_18_ column (150 mm × 4.6 mm internal diameter, 5 µm particle size) that was purchased from ACE, United Kingdom. The optimum mobile phase was prepared by mixing methanol, acetonitrile, and water in the ratio of 40:40:20 (by volume). The mobile phase was filtered by using a 0.45 μn microporous filter and was degassed by sonication prior to use. A wavelength of 260 nm was chosen since it was found to be the most appropriate for the determination of the two active ingredients because both of the drugs had sufficient absorption at this wavelength. The flow rate used was 0.5 ml/minute. The injection volume was 20 μl and the temperature of the column was 40°C. The total run time of the system was about 10 minutes.

### Preparation of Standard Solution

The standard solution for both drugs was prepared by dissolving 50 mg AZL and OLM reference standards into 50 ml of methanol in two separate 100-ml volumetric flasks. Final volumes were adjusted with methanol to prepare 500 µg/ml standard stock solutions of both drugs. Using a volumetric pipette, 20 ml of this solution was transferred to a 100 ml volumetric flask and completed to the volume using the mobile phase. The obtained final solution contained 100 μg/ml of AZL and OLM.

### Preparation of Sample Solution

Twenty tablets were weighed and powdered. An accurately weighed quantity of the powder equivalent to 16 mg of Azelnidipine and 20 mg of Olmesartan was taken into a 100-ml measuring flask and dissolved in 50 ml methanol with sonication. The solution was filtered through 0.45 µm membrane filter and the residues were washed thoroughly with methanol. The filtrate and washings were combined in a 100-ml volumetric flask and diluted to the mark with methanol to get a final concentration of 160 µg/ml of Azelnidipine and 200 µg/ml of Olmesartan. For the final test solution of Azelnidipine and Olmesartan, 1.0 ml of filtrate of the sample solution was transferred to 10-ml volumetric flasks and diluted up to the mark with the mobile phase.

### Forced Degradation Study

ICH prescribed stress conditions such as acidic, basic, oxidative, thermal (solid heat), and photolytic stresses, which were carried out.

### Standard Drug Stock Solutions

Forced degradation studies of both of the drugs were carried out under the conditions of hydrolysis, oxidation, and photolysis. An accurately weighed quantity of the powder, 160 mg of Azelnidipine and 200 mg of Olmesartan, was taken into a 50-ml measuring flask and diluted to the mark with the mobile phase to get a final concentration of 3200 µg/ml of Azelnidipine and 4000 µg/ml of Olmesartan. These stock solutions were used for the forced degradation study.

### Acid Hydrolysis

Forced degradation in acidic media was performed by taking 10 ml of the stock solutions of Azelnidipine and Olmesartan, each in separate amber round-bottom flasks. Then 10 ml of 1 N HCl was added and these mixtures were kept at reflux for 4 hours. This solution was neutralized with 1 N NaOH before analysis.

### Base Hydrolysis

Forced degradation in basic media was performed by taking 10 ml of stock solutions of Azelnidipine and Olmesartan, each in separate amber round-bottom flasks. Then 10 ml of 1 N NaOH was added and these mixtures were kept at RT for 6 hours. This solution was neutralized with 1 N HCL before analysis.

### Oxidative Hydrolysis

Degradation with hydrogen peroxide was performed by taking 10 ml of stock solutions of Azelnidipine and Olmesartan, each in two different flasks, and adding 10 ml of 3% (w/v) hydrogen peroxide in each of the flasks. These mixtures were kept for up to 3 days in the dark.

### Thermal Degradation

To study neutral degradation, 10 ml of stock solutions of Azelnidipine and Olmesartan were taken in two different flasks, then 10 ml of HPLC grade water was added in each flask, and these mixtures were heated for 12 hr at reflux in the dark.

### Photodegradation

The photostability was studied by exposing the solid state of both the drugs to direct sunlight in summer days for 6 hr on a wooden plank.

For HPLC analysis, all the degraded sample solutions were diluted with mobile phase to obtain the final concentrations of 32 μg/ml of Azelnidipine and 40 μg/ml of Olmesartan. Besides, the solutions containing 32 μg/ml of Azelnidipine and 40 μg/ml of Olmesartan were also prepared separately without performing the degradation of both of the drugs. Then the 20 μl solution of the above solutions were injected into the HPLC system and analyzed under the chromatographic analysis condition described earlier.

### Forced Degradation Study on the Sample Solution

Twenty tablets were weighed and powdered. An accurately weighed quantity of the powder equivalent to 160 mg of Azelnidipine and 200 mg of Olmesartan was taken into a 100-ml measuring flask and dissolved in the mobile phase with sonication for 20 minutes. The solution was filtered through a 0.45 µm membrane filter and the residues were washed thoroughly with mobile phase. The filtrate and washings were combined in a 50 ml volumetric flask and diluted to the mark with mobile phase to get a final concentration of 3200 µg/ml of Azelnidipine and 4000 µg/ml of Olmesartan.

The following conditions for the forced degradation studies were the same as that used for the standard solution. After that, the above solutions were diluted with the mobile phase to get a final concentration of 32 μg/ml of Azelnidipine and 40 μg/ml of Olmesartan. The 20 μl solutions of the above solutions were injected into the HPLC system and analyzed under the chromatographic analysis conditions described earlier.

## Conclusion

The validated HPLC method developed for the quantitative quality control determination of AZL and OLM in combination was evaluated for system suitability, specificity, sensitivity, linearity, range, accuracy (recovery), precision (repeatability and intermediate precision), and robustness. All the validation results were within the allowed specifications of ICH guidelines. The developed method has proven to be rapid, accurate, and stability-indicating for the simultaneous determination of combined AZL and OLM in tablet dosage form in the presence of excipients and the degradation products. There was always a complete separation of both ingredients from their degradation products and from the placebo. As a result, the proposed HPLC method could be adopted for the quantitative quality control and routine analysis of the tablet dosage form or any other formulation.
